# How Neuronal Noises Influence the Spiking Neural Networks’s Cognitive Learning Process: A Preliminary Study

**DOI:** 10.3390/brainsci11020153

**Published:** 2021-01-25

**Authors:** Jing Liu, Xu Yang, Yimeng Zhu, Yunlin Lei, Jian Cai, Miao Wang, Ziyi Huan, Xialv Lin

**Affiliations:** School of Computer Science and Technology, Beijing Institute of Technology, Beijing 100081, China; jing.liu-22@hotmail.com (J.L.); 2220170740@bit.edu.cn (Y.Z.); 3120201035@bit.edu.cn (Y.L.); 3120201001@bit.edu.cn (J.C.); 3220201093@bit.edu.cn (M.W.); 3220200891@bit.edu.cn (Z.H.); 3220201066@bit.edu.cn (X.L.)

**Keywords:** spiking neural network, neuronal noise, cognitive function

## Abstract

In neuroscience, the Default Mode Network (DMN), also known as the default network or the default-state network, is a large-scale brain network known to have highly correlated activities that are distinct from other networks in the brain. Many studies have revealed that DMNs can influence other cognitive functions to some extent. This paper is motivated by this idea and intends to further explore on how DMNs could help Spiking Neural Networks (SNNs) on image classification problems through an experimental study. The approach emphasizes the bionic meaning on model selection and parameters settings. For modeling, we select Leaky Integrate-and-Fire (LIF) as the neuron model, Additive White Gaussian Noise (AWGN) as the input DMN, and design the learning algorithm based on Spike-Timing-Dependent Plasticity (STDP). Then, we experiment on a two-layer SNN to evaluate the influence of DMN on classification accuracy, and on a three-layer SNN to examine the influence of DMN on structure evolution, where the results both appear positive. Finally, we discuss possible directions for future works.

## 1. Introduction

The human brain is a very sophisticated system. Billions of neurons form into a huge network. The human brain is capable of dealing with many different kinds of functions in parallel. Spiking signal trains are sent from different sources carrying different kinds of information roaming in that network.

Roughly 20 to 40% of the total energy is consumed by the brain handling tasks, while the rest of the energy, identified by Raichle as the dark energy [1], is used for background activities. Form the viewpoint of cognitive functions, spiking signal trains employed by other background functions could be considered as neuronal noises. Moreover, it would be reasonable to assume that the combination or overlapping of so many spiking signal trains could be expressed as continuous additive white noise. Further evidence might be that many brain science researches have also demonstrated that sometimes quantitative EEG in human brain could also be approximated as white noise. Existing findings in neuroscience and cognition science indicate that the dark energy possibly has connections with cognitive functions, which presented us with the idea of simulating the dark energy environment for SNNs and investigating whether it helps information processing in a computer science problem.

In a resting brain, in contrast to people’s normal perception of silence, spontaneous neural fluctuations can still be observed. Early research introduced the idea that, if the ElectroEncephaloGraphy (EEG) were successfully applied in human brains, spontaneous ongoing electrical signals could be recorded [2]. This indicates that, even when people fall asleep, the neurons are still working. Scientists later defined the brain regions that involved in activities of the rest state as a Resting State Network (RSN). The widely-recognized model of RSNs is DMN [3]. Typical DMNs are studied under unconsciousness or with minimal consciousness, including but not limited to sleep, anaesthesia, and coma, and this brings neural researches to cognitive science.

To date, researchers have been interested in discovering how DMNs can affect cognitive functions. Preexisting findings give evidence that there are correlations between the DMN and the cognition behavior. An experiment was conducted to find the associations and dissociations of DMN and self-reference network [4]. It proved that functional associations (rest and self-reference) existed between the two networks, both activated in some same cerebral regions, i.e., the medial PreFrontal Cortex (mPFC), and the Posterior Cingulate Cortex (PCC)/precuneous. Another recent study which aimed to find changes of DMN complexity in the brains of patients with Alzheimer Disease (AD) [5] found that cognitive decline had a positive correlation with the reduction of signal complexity in DMN. These studies imply that DMN is positively related to the cognition system.

Brain science researches also show that Default Mode Network (DMN) is strongly related to cognitive functions. According to research, DMN changes with age. It has been shown that there is limited evidence of DMN in infancy but that DMN connectivity can be detected in children from 9 to 12 years of age. By adulthood, the network shows the most stable state. By old age, the connection of the network degrades significantly due to the decline of cognitive function. Therefore, the change of the DMN reflects the tissue pattern of spontaneous activity of the cerebral neural system, which may be related to the learning, memory, and other cognitive functions of the brain. However, the mechanism of all this remains unrevealed.

SNNs can handle nonlinear problems because of the spatio-temporal properties they learnt from the bio-neural system. The spatial feature comes from the interconnections of neurons via dendrites, and even axons, to a broader extent. The temporal feature comes with the spike trains, where spikes arriving on a neuron differ from time.

Thus, in this work, we try to use additive white noise to mimic an initialized Default Mode Network’s influence on cognitive functions. Taken together, specifically, we apply SNN to solve a cognitive problem, and compare the accuracy with and without DMN affecting the SNN, in addition to observing synaptic growths of the SNN.

In Section 1, a brief background of DMN and SNN is given respectively, and the motivation to apply those neuronal signals in an SNN is introduced. The method and models are covered in Section 2. Section 3 explains the experiments and illustrates the result. A discussion of the result and possible directions for future works are given in Section 4.

## 2. Methods and Models

In this section, the experimental method is first introduced, along with the models and materials comprising the method. The model selection intends to simulate the neural information processing in a bionic environment to the greatest extent, apart from the consideration of implementation complexity.

The experiment consists of two parts, one is an assessment of the influence of DMN on prediction accuracy of a two-layer network, the other is an observation of the influence of DMN on structure evolution (synaptic growths) in the memory layer of a three-layer network. Primarily, we choose SNN for networking according to its bionic properties. The detailed topology design is covered in Section 3. Besides, the two experiments require a neuron model and a noise model in common. The neuron model is the basic element of SNN to comprise the topology, and the noise is used to simulate the DMN and added to the SNN. Then, an encoding scheme is discussed. It is applied in the network to transform pixel information into signals, as informative inputs are fed into the neurons. Finally, a learning algorithm that helps the network forming the memory, along with its formulaic representation is given.

The experiment aims to explore how background noise could help cognition. Thus, we employ an image classification problem for the evaluation of the method. For materials and tools, we choose MNIST [6] as the dataset, providing patterns of written digits, and experiment upon NEST (the Neural Simulation Technology Initiative) simulator [7].

### 2.1. The Neuron Model: Leaky Integrate-and-Fire

For computational convenience, SNNs are prone to utilize electrical neuron models rather than pharmacological models. The two popular electrical neuron models are Integrate-and-Fire (IF) and Hodgkin-Huxley (HH), where the number of parameters to achieve an accurate HH model addresses a very high computational complexity, which is not adaptive to a typical ANN (Artificial Neural Network) learning processes. Hence, IF is more applicable in the ANN domain. IF was firstly investigated by Lapicque in 1907 [8,9]; however, it has a limitation of no temporal memory supported. Later, the LIF (Leaky Integrate-and-Fire) model, derived from IF model, was introduced, which resolved that problem.

**Figure 1:**
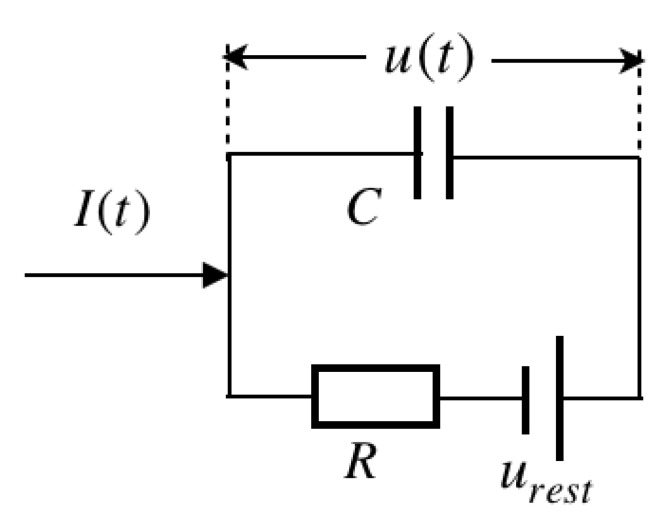
A circuit representation of the electrical properties of a cell membrane.

A basic LIF model consists of (a) a linear differential equation and (b) a spike firing threshold [10]. The equivalent circuit of the cell membrane, as illustrated in Figure 1.

The common form of the linear differential equation can be deduced as

(1) $$\tau_{m}\frac{du}{dt}=-\left[u\left(t\right)-u_{rest}\right]+RI\left(t\right)$$

where $\tau_{m}=RC$ is the membrane time constant, $u_{rest}$ is the resting potential, and I(t) is the driving current. Specifically, $R=1/g_{l}$, where $g_{l}$ is the leak conductance.

**Figure 2:**
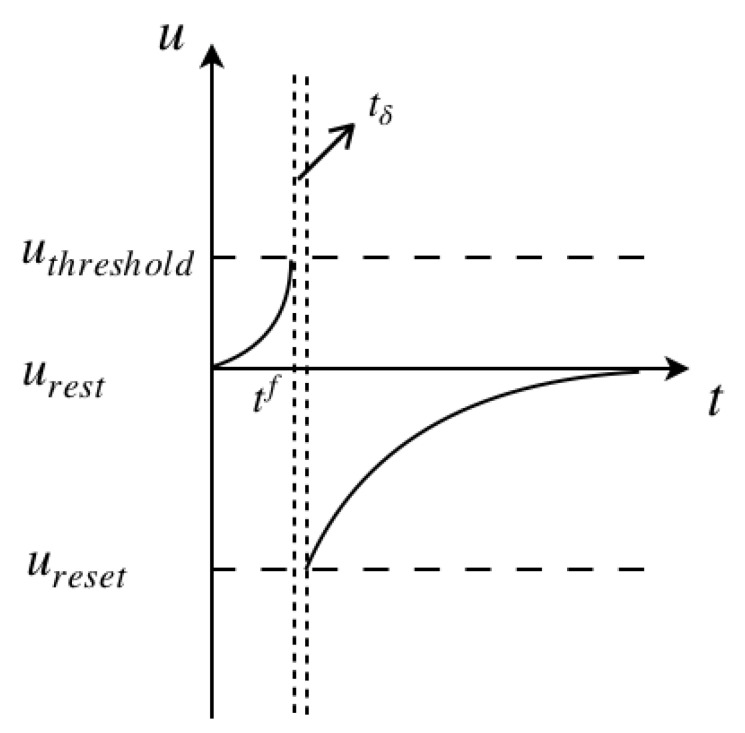
Changes of the membrane potential with a spiking impulse acting on a Leaky Integrate-and-Fire (LIF) model.

The firing threshold is given by

(2) $$\lim_{\delta \to 0;\delta >0}u\left(t^{f}+t_{\delta }\right)=u_{reset}$$

where $t^{f}$ denotes for the firing time, in particular, superscript f=1,2,…,n refers to the number of the spike. Figure 2 illustrates the process of the membrane potential change with a spiking pulse acting on it. The initial state of the cell membrane is assumed at the rest potential, and the neuron is fired at $t^{f}$, the intersection of the left vertical dashed line and the *t* axis. After a very small time gap $t_{\delta }$, shown between the two vertical dashed lines, the membrane potential drops down to the reset potential, later recovering back to the rest potential.

### 2.2. DMN Model: Additive White Gaussian Noise

We simulate the DMN by adding noise that down to the neuronal scale. For a neuron membrane, noises come mainly from three sources: (**a**) thermal noise, (**b**) ionic channel noise, and (**c**) stochastic synaptic activities [11]. The first two noise sources are classified as intrinsic noise, while the last one is as extrinsic noise. The intrinsic noise is produced during neuronal intrinsic activities. The extrinsic noise, however, is caused by activities from other neurons or network errors. Thermal noise is white noise and is relatively weaker than other noise sources. Ionic channel noise caused by conductance fluctuations can be either modeled as either multiplicative or additive, depending on the modeling approach. For synaptic activities, it plays the dominant role and can be modeled as AWGN.

As an initial attempt, we added the noise source that has the major importance, i.e., synaptic activities, to the LIF model.

AWGN yields three characteristics, additive, white, and Gaussian. Its additive property means it can be simply added to the original signal. For the white characteristic, the noise is uniformly distributed across the frequency band. For the Gaussian feature, it has a normal distribution with zero mean in the time domain.

Taking the noise into account, the LIF differential equation then is modified into a stochastic differential equation, which also is known as the Langevin equation. The equation is given below [12,13],

(3) $$\tau_{m}\frac{du}{dt}=-\left[u\left(t\right)-u_{rest}\right]+RI\left(t\right)+\xi \left(t\right)$$

where $\xi \left(t\right)=RI_{sto}\left(t\right)$ is a stochastic voltage input, representing the Gaussian white noise.

The stochastic current $I_{sto}\left(t\right)$ given by the NEST Gaussian noise generator is

(4) $$I_{sto}\left(t\right)=\mu +\sigma t^{f}$$

where μ stands for the mean of the amplitude of the noise current, σ represents the standard derivation, and $t^{f}$ is the time label, in which superscript f=1,2,…,n.

### 2.3. Spikes Encoding

It is convincible that, for a greyscale image, pixels with higher value are more informative. From this perspective, to convert each pixel into a spike signal, we designed our spiking encoding method comply with the idea of Rank Order Coding (ROC) [14]. In general, the idea is to convert the RGB value [0, 255] of a pixel into the time that a spike arrives at a neuron, claiming that the earlier the spike arrives, the more important it is. A related study [15] explored the effectiveness of the transformation from pixels to temporal information with different encoding schemes, including linear function, exponential function, inverse function, and power function. A comparison is given in a two-dimensional figure with a pixel’s RGB value as the x-axis, and the converted arrival time as the y-axis. The exponential and the inverse encoding scheme both yield a steep decline when the RGB rises from 0, but slows down at the end. This is not ideal for the situation because it shows no obvious difference of the arrival time when pixels grow to be more informative. In contrast, the linear function and the exponential function are the right candidates for the encoding scheme. Amongst the two, we select the exponential function. The spike time $S_{k}$ of a pixel *k* with its RGB value $V_{k}$ is converted within time interval $\left[T_{start},T_{stop}\right]$

(5) $$S_{k}={\left(V_{k}-1\right)}^{2}\times \left(T_{stop}-T_{start}\right)+T_{start}$$

### 2.4. The Learning Algorithm

As we evaluate the hypothesis on an image classification problem, which in terms of cognition is regarded as a perceptual decision making process, we would select an approach to approximate neuronal activities that comprise the process. A typical cognitive decision-making process contains three major steps. First, the inputs and outputs should be present with weights on each match. Next, we select a match based on the input and the weight. Then, we assess the result and possibly give feedback to enhance the correctness of the selection. The whole process can be looped for several times to achieve a better outcome.

This process, in traditional Convolutional Neural Networks (CNNs), is implemented with statistical approaches, for instance, backpropagation. However, it does not reveal the causality of the input and the result. Scaling down to neural networks, connections among neurons can be learnt causally.

The process of forming the memory is denoted with its neuroscientific meaning explained by Hebbian theory [16]. It is described as follows. Assume two neurons A and B are spatially near enough, if B’s excitation is observed with A’s persistently, then it is believed that A involves in exciting B. Derived from the Hebbian theory, a little modification on the temporal feature stating that if B’s trigger is found right after A’s, leads to the concept of STDP.

STDP shows that, to adjust the intensity of synaptic connectivity of two neurons, the presynaptic neuron can either strengthen, also known as Long-Term Potentiation (LTP), or weaken, as Long-Term Depression (LTD), the postsynaptic neuron. STDP thereby explains the mechanism of forming the memory in cellular scale. To help our networks with memorizing, we designed a supervised learning approach based on STDP. If an output matches the input, the intensity of the synaptic connection, reflecting its memory, then increases; otherwise, if the causality is wrong, the intensity is weakened.

We use weight change Δw of a synapse *i* to represent the adjustment of synaptic intensity. Synapse *i* of the presynaptic neuron *pre* connects with the postsynaptic neuron *post*. The time that the presynaptic stimuli arrives at the synapse *i* is denoted as $t_{pre}^{a}$, where a=1,2,…,n. $t_{post}^{b}$ represents the time that the spike excites the postsynaptic neuron, where b=1,2,…,n. The weight change is thus represented as [17]

(6) $$\Delta w_{i}=\sum_{a=1}^{N}\sum_{b=1}^{N}f\left(t_{post}^{b}-t_{pre}^{a}\right)$$

where f(Δt) is the weight adjustment function. There are many different variations of STDP rules. In this study, we designed our method based on a simple but widely-used STDP model (Song, Miller & Abbott, 2000). It is also known as the pair-based STDP model. Define $\Delta t=t_{post}-t_{pre}$, so that

(7) $$f\left(\Delta t\right)=\left\{\begin{array}{cc} A_{+}\exp\left(-\Delta t/\tau_{+}\right) & \mathrm{if}\;\Delta t>0\\ -A_{-}\exp\left(\Delta t/\tau_{-}\right) & \mathrm{if}\;\Delta t<0 \end{array}\right.$$

where $A_{\pm }$ stands for the amplitude of changes on the synaptic weight, and $\tau_{\pm }$ denotes for the membrane time constant. For f(Δt)>0, it denotes for a LTP process; otherwise, for f(Δt)<0, it represents an LTD.

Taken together, we apply AWGN as the cerebral background noise, encode pixel information to temporal spikes as inputs to neurons, employ STDP to form memory and use supervised learning to train the networks. The detailed experimental process is given in Section 3.

## 3. Experiments

In this section, we describe the design, the process, and the result of the two-layer network and three-layer network respectively. Experiments are designed based on the hypothesis that the engagement of DMN (represented as neuronal noise in this paper) has positive impacts on SNN processing information. Beforehand, we first explore the variables in the Gaussian white noise model, where the characteristics are considered in the design of the two experiments later on.

### 3.1. Variables in the Gaussian White Noise Model

Using noise generators are not new in neuronal experiments. In early studies, researchers added white noise and colored noise to simulate input spiking trains [18], but not the noise itself.

**Figure 3:**
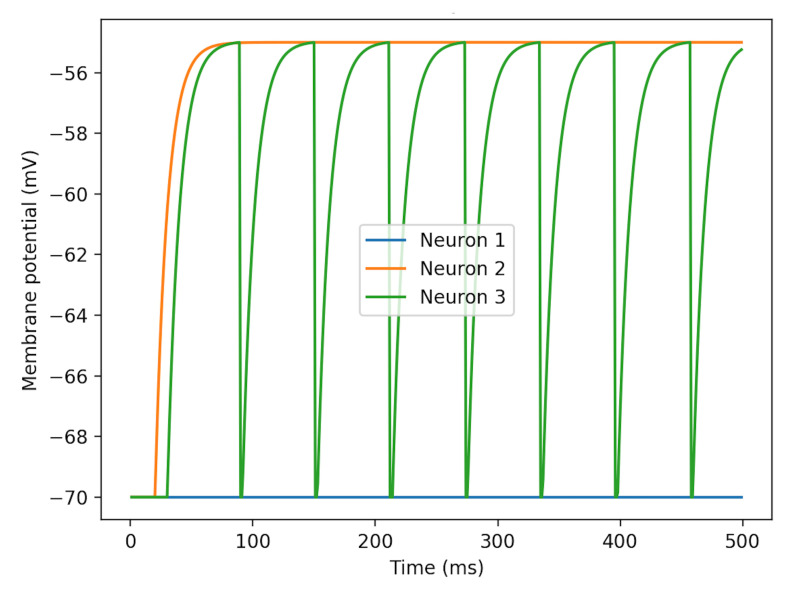
Patterns of membrane potentials of three LIF Neurons differing with μ in the Neural Simulation Technology Initiative (NEST) Gaussian white noise generator. Neuron 1 has μ=0, neuron 2’s μ=375 pA and neuron 3 is excited at μ=376 pA. σ is set to the default as 0 for all the noise generators. The figure demonstrates that the firing potential is in the range of (375, 376] pA.

NEST provides an approximation of Gaussian white noise. Recalling from Equation (4), the noise generator provides variables μ and σ, both with a default value of 0 pA. First, keep σ default and set μ as the single variable. Without neuronal spike inputs, patterns of membrane potentials changing with μ are illustrated in Figure 3. Neuron 3’s curve demonstrates the process of charging and discharging. The rest potential is at −70 mV and the fired potential is at about −55 mV.

**Figure 4:**
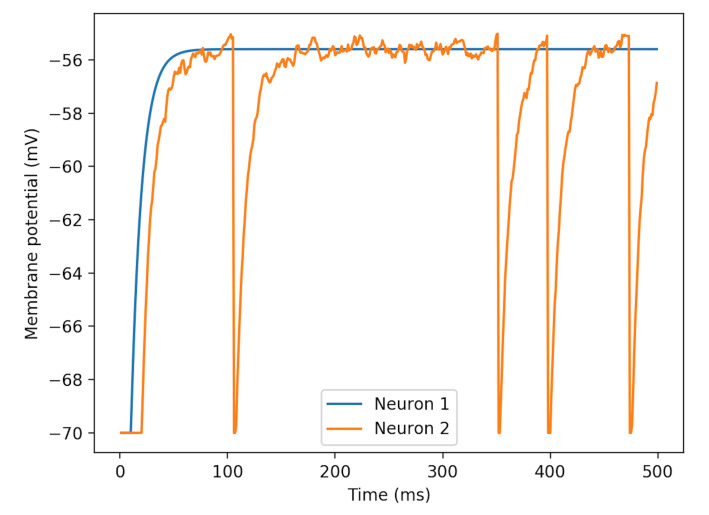
Patterns of membrane potentials of three LIF neurons differing with σ in NEST Gaussian white noise generator. Neuron 1, 2 has σ=0,40 pA respectively, where μ=360 pA is set to both. The curve of neuron 2 fluctuates upon the curve of neuron 1.

Based on the result from above, we set μ close to the firing potential, and change σ slightly. As is shown in Figure 4, σ adds randomness on the noise.

### 3.2. Assessment on the Classification Accuracy of a Two-Layer Network

**Figure 5:**
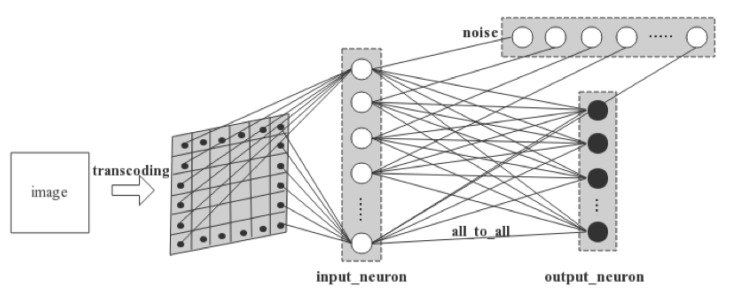
The model of the two-layer network.

The two-layer network involves an input layer and an output layer. As is illustrated in Figure 5, a 28 × 28 MNIST image is first converted into spiking signals by pixel. Each pixel’s RGB value is converted into the temporal information carried by a spike and is conveyed to a LIF neuron in the input layer in a one-to-one manner. Thus, there are in total 28 × 28 = 784 neurons that consist of the input layer. Next, the input neurons are connected one-to-one with Gaussian white noise generators. Finally, the output neurons are fully connected with the input neurons. We set 10 neurons in the output layer as there are 10 digits, i.e., 0–9 s, to be recognized in MNIST dataset.

**Figure 6:**
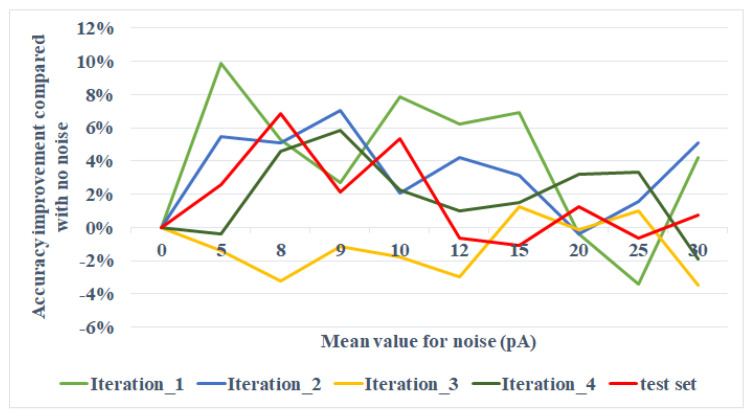
The accuracy-mean line chart. *Iteration_n* in the legend stands for a single training process iterated for *n* times.

We use the default values of LIF parameters, as is listed in Table 1, and turn only the mean of the noise, keeping the standard deviation as default. The experiment result is shown in Figure 6. For the testing set, the accuracy improvement compared with the situation where no noise is added reaches at peak at the mean around 8 pA. The result is discussed in Section 4.

Moreover, we add a preprocessing step before spiking encoding. The raw image is processed with a 4×4 convolutional filter with stride as 1, and downsampled with a 2×2 pooling layer. After transformation, the resolution of the image becomes 12×12. The rest operations are the same as the aforementioned operations. However, in this attempt, the result yields no obvious difference when turning the mean of the noise.

### 3.3. Observation on Structure Evolution of a Three-Layer Network

In our opinion, SNN’s great advantage lies in its adaptive structure changing ability, where synapses would grow or degrade on demand. In SNN, according to biological research, Hebb’s rule is used to guide the growth of new synapses in the network along the structure evolution process as a reflection of external stimulation. In addition to the input and output layers, the three-layer network adds an intermediate memory layer. Experiments upon the three-layer network are conducted in the order of (a) observing synaptic growths with image input and no noise acting on the neurons in the memory layer; (b) observing synaptic growths with no image input and only random noise acting on neurons in the memory layer; and (c) observing synaptic growths with both image input and noise acting on neurons in the memory layer. The first experiment mimics the situation where DMN is not involved, and only learning from external stimulations. The second experiment mimics the situation where some preliminary DMN is involved to form original knowledge (expressed by some original synapses generated under random noise) before learning from external stimulation. Since it is known that when the noise input is under the firing potential, the neuron would not be excited, and there would be no present growths, the amplitude of noise current is set with μ=400 pA and σ=0.

During the second experiment, with the absence of the input layer, when roughly 20% of the neurons have noise added on, the scale of synaptic growths reaches a proper scale. Thus, 120 noise generators are linked with randomly chosen memory neurons one-to-one.

**Figure 7:**
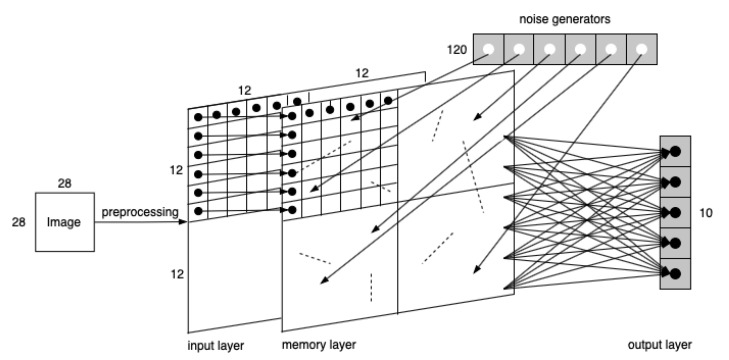
The model of the three-layer network. Dashed lines represent preexisting synaptic connections among neurons.

For this set of experiments, where the model is demonstrated in Figure 7, an MNIST image is processed through four 4×4 convolutional filters with stride as 1, and then a 2×2 pooling layer in sequence yields four 12×12 transformed layers. Each pixel then is encoded into a temporal spike signal that enters the corresponding neuron in the memory layer. The output neurons are fully connected with memory neurons.

**Figure 8:**
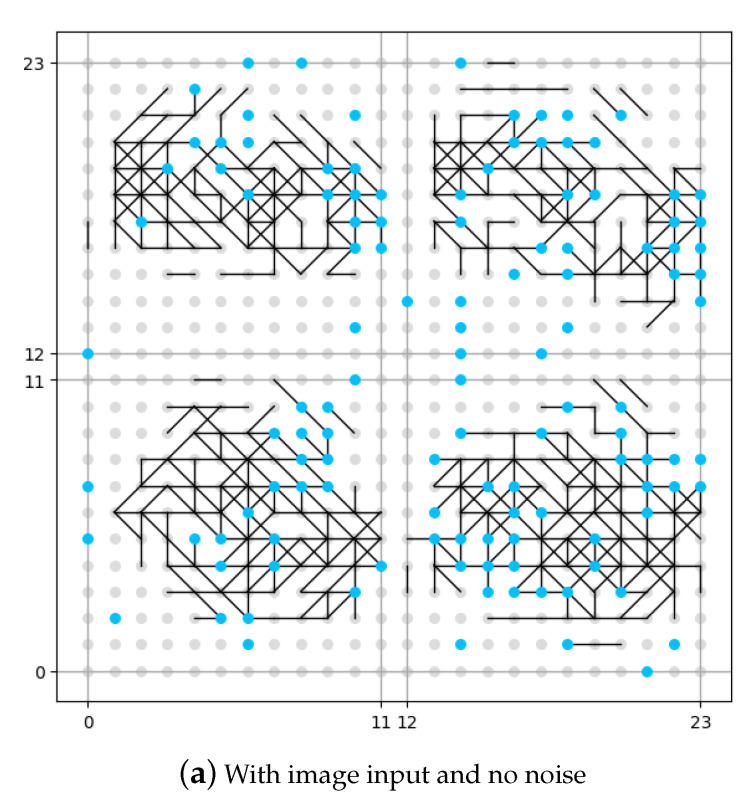

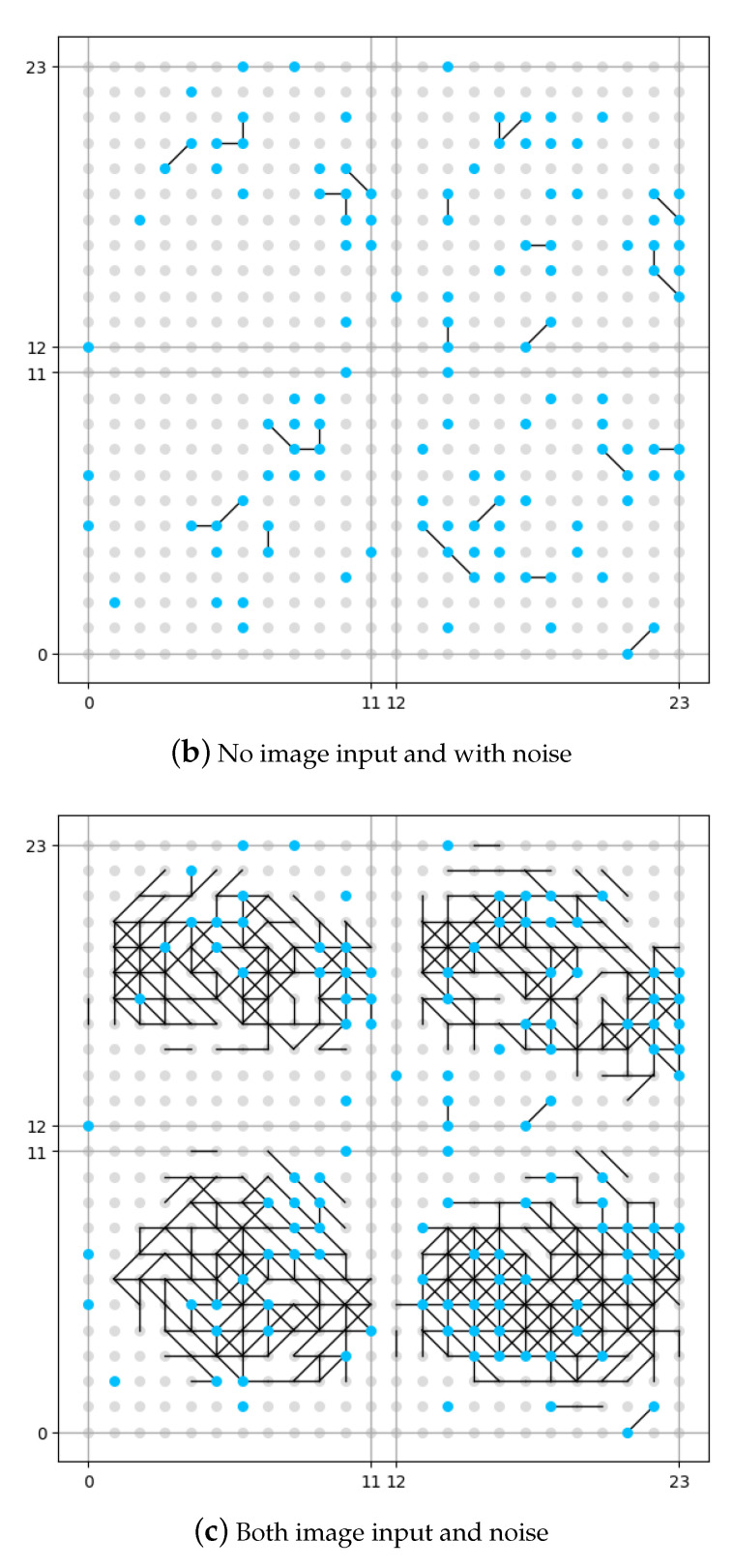
The comparison of synaptic growths observed in the memory layer.

The result of this set of experiments is shown in Figure 8. The neuron in the memory layer that has been connected to a noise generator is identified as a blue dot. The neuron in the memory layer that has not been connected to a noise generator is identified as a grey dot. In order to make a better image for comparison, although in the first experiment (as shown in Figure 8a) there is no noise input, we have still identified the position of neurons connected to noise generators, but be aware that no noise is added. The vertical and horizontal axes in the figure are the coordinates for the neurons in the memory layer.

It could be seen from Figure 8b that if no image is input, the memory layer would generate some original synapses under the influence of DMN (expressed as noise in this work), like the pre-set knowledge. From the comparison between Figure 8a and Figure 8c we see that, if no DMN is involved (Figure 8a), the memory layer would generate new synpases under the external stimulation (input image), but is rather sparse compared with the situation where DMN is involved (Figure 8c). The number of synapses under the situation where DMN is involved is roughly 20% more that the number of synapses under the situation where DMN is not involved according to our experiment. We could draw the conclusion that the presence of DMN (expressed as neural noise in this work) can make the synaptic learning of the spiking neural network fuller. More growth of new synapses could mean more information is recognized and stored by the network, which might also indicate more robustness.

## 4. Discussion

In this paper, we introduce two experiments to explore how neuronal noises help SNNs. The two-layer network aims to examine the accuracy with a little noise added on, while for the three-layer network, we observe the nature of synaptic growths when the noise reaches at or beyond the firing potential of LIF neurons.

For the two-layer network experiment, we turned μ in the range of (0, 30] pA and observed a local peak of accuracy improvement at around 8 pA, as shown in Figure 6. The next step is to see a general tendency when the range of μ expends from 0 to the value that could fire the neurons. The comparison of the trends beneath and above the threshold could be interesting for further study. Afterwards, we experiment on processed images. Data preprocessing has both its bionic and computational significance. As cerebral neurons ignore signals beneath some thresholds, we conduct data preprocessing to help the neural network complete this step when simulating with ANNs. This process reduces the computational complexity, especially for medium- or large-scale networks. In this study, for example, white pixels carry little information, so that those pixels are tailored before transmitting to the input neurons. However, an interesting phenomenon is that when the image is preprocessed, the accuracy yields no obvious difference. From the perspective of Signal-to-Noise Ratio (SNR), it can be explained that the encoded spike of the processed image with a resolution of 12×12 over the noise is close to 1, or even the noise predominates when the amplitude of current rises. Research on SNR for homologous problems is another aspect.

For the three-layer network experiment, it is obvious that when neurons try to memorize the pattern of an image, they leave traces, i.e., they form new synaptic links. From the result, we may infer that the existence of DMN (expressed as neuronal noise) helps to form some original connections among neurons, which might be viewed as pre-set knowledge. When an external stimulus comes, the new trace that would be created may have overlaps with preexisting connections, so that the memory could be formed faster than it is expected to be (without neuronal noise).

Additionally, neurons cannot be triggered by weak spikes, so that neuronal noises might be additive to reinforce these input spikes. If a summed signal has the intensity beyond the firing potential, the primary spike turns out to be valuable. We only analyze additive noise here; however, there might be other types of noises existing in cerebral cortices, for example, multiplicative noises. Its appearance depends on the quality of the communication channel that the signal propagates in. Thus, in this case, the noise could not be simply added to the signal and consequently, the statement above would not be true. Besides, colors of the noises is another respect that could matter. In our study, we use white noise. The pink noise, or 1/f noise, however, is believed to be the most common noise in vivo [19]. A possible direction for further study is to examine how these noises, that are proven to be present in a brain, could affect differently in simulation. It may help neuroscientists gain a better understanding of the engagement of neuronal noises in cognitive processes.

In terms of SNNs, it is worth discovering relations among network scales, type and value of noises, and other relevant factors, so that neuronal noises could enhance the performance of SNNs, with regards to accuracy, adaptabilities, and speed, etc.

## 5. Conclusions

We have presented our work to use additive white noise to mimic an initialized Default Mode Network’s influence on cognitive functions in this paper. For two-layer SNN, our experiments show that neuronal noises within certain ranges would help to enhance the cognitive learning ability of the SNN, which might also be proved helpful for other SNN learning algorithms. For three-layer SNN, the presence of DMN (expressed as neuronal noises) would provoke the memory layer of SNN to grow original synaptic connections to represent some pre-set knowledge. The presence of DMN (expressed as neuronal noises) would also enhance the synaptic learning process of the SNN to make it grow more new synapses. In the next step of our work, we will explore how the cognitive functions would in turn influence the behavior of the DMN.

Our experiment results with MNIST data-set can show the trend of the noises’ influence on the leaning process of SNN. In the future, we could explore the employment of neuronal noise to help establish more powerful algorithm on classification of MNIST, which is beyond the scope of this work. Also, here we use white noise to mimic DMN in an initialized state to see the influence on the learning process of SNN. If the DMN has already been loaded with pre-learned knowledge, we believe that the learning ability would be somewhat enhanced.

## Figures and Tables

**Table 1:** Major values by default of LIF neuron model parameters in NEST.

Parameter	Value	Explanation
$V_{m}$	Null	Membrane potential
$E_{L}$	−70.0 mV	Resting potential
$V_{\mathrm{th}}$	−55.0 mV	Spike threshold
$V_{\mathrm{reset}}$	−70.0 mV	Reset potential
$C_{m}$	250.0 pF	Capacity of the membrane
$\tau_{m}$	10.0 ms	Membrane time constant

## Data Availability

The MNIST data-set is an opensource data-set.
